# Comprehensive Study and Design of Graphene Transistor

**DOI:** 10.3390/mi15030406

**Published:** 2024-03-18

**Authors:** Qian Cai, Jiachi Ye, Belal Jahannia, Hao Wang, Chandraman Patil, Rasul Al Foysal Redoy, Abdulrahman Sidam, Sinan Sameer, Sultan Aljohani, Muhammed Umer, Aseel Alsulami, Essa Shibli, Bassim Arkook, Yas Al-Hadeethi, Hamed Dalir, Elham Heidari

**Affiliations:** 1 Department of Electrical and Computer Engineering, Malachowsky Hall, University of Florida, Gainesville, FL 32611, USA; qian.cai@ufl.edu (Q.C.); jiachi.ye@ufl.edu (J.Y.); b.jahannia@ufl.edu (B.J.); hwang40@ufl.edu (H.W.); c.patil@ufl.edu (C.P.); hamed.dalir@ufl.edu (H.D.); 2 Department of Physics, Faculty of Science, King Abdulaziz University, Jeddah 21589, Saudi Arabia; rredoy@stu.kau.edu.sa (R.A.F.R.); asidam0004@stu.kau.edu.sa (A.S.); cnans1995@gmail.com (S.S.); saljohani0203@stu.kau.edu.sa (S.A.); mumer@stu.kau.edu.sa (M.U.); aalsulami1444@stu.kau.edu.sa (A.A.); eshibli@kau.edu.sa (E.S.); barkook@kau.edu.sa (B.A.); yalhadeethi@kau.edu.sa (Y.A.-H.)

**Keywords:** graphene, field effect transistor, 2D material, chemical vapor deposition

## Abstract

Graphene, renowned for its exceptional electrical, optical, and mechanical properties, takes center stage in the realm of next-generation electronics. In this paper, we provide a thorough investigation into the comprehensive fabrication process of graphene field-effect transistors. Recognizing the pivotal role graphene quality plays in determining device performance, we explore many techniques and metrological methods to assess and ensure the superior quality of graphene layers. In addition, we delve into the intricate nuances of doping graphene and examine its effects on electronic properties. We uncover the transformative impact these dopants have on the charge carrier concentration, bandgap, and overall device performance. By amalgamating these critical facets of graphene field-effect transistors fabrication and analysis, this study offers a holistic understanding for researchers and engineers aiming to optimize the performance of graphene-based electronic devices.

## 1. Introduction

**Figure 1:**
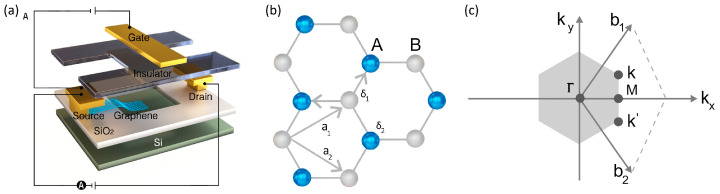
(**a**,**b**) A typical GFET structure showcases a graphene layer as the electron flow channel atop an insulating gate dielectric layer, with efficiency and leakage risks influenced by the dielectric’s thickness. Mounted on a silicon or silicon dioxide substrate, graphene’s carbon atoms form a honeycomb pattern with sp^2^ hybridization, featuring lattice vectors of approximately 2.46 Å at 120° angles. (**c**) represents the reciprocal lattice of graphene, illustrating the first Brillouin zone with high-symmetry points labeled Γ, *K*, and *K*′. The hexagonal shape corresponds to the symmetry of the graphene lattice in reciprocal space. The points *K* and *K*′ are the corners of the Brillouin zone, known as the Dirac points, where the conduction and valence bands of graphene touch and exhibit linear dispersion, leading to graphene’s unique electronic properties. The vectors *b*1 and *b*2 would be the reciprocal lattice vectors.

Graphene has emerged as a promising material for next-generation electronics due to its exceptional electrical, optical, and mechanical properties [1]. It possesses a honeycomb lattice structure of graphene, and its hexagonal shape corresponds to the symmetry of the graphene lattice in reciprocal space, as shown in Figure 1b,c. Specifically, graphene field-effect transistors (GFETs) have demonstrated the potential to outperform traditional silicon transistors in various applications such as flexible electronics, high-frequency devices, and sensors [2]. However, realizing commercially viable GFETs requires overcoming significant fabrication challenges related to scalability, reproducibility, and cost-effectiveness [3,4,5,6]. This literature review provides a timely and in-depth analysis of state-of-the-art GFET fabrication processes and methodologies, focusing on addressing these aforementioned challenges through advanced materials engineering techniques and manufacturing approaches.

We systematically scrutinize critical facets of GFET development including graphene synthesis, transfer, and metrological assessments of quality. Recognizing that graphene’s pristine condition profoundly impacts device performance, we explore techniques to preserve superior electrical, structural, and quantum transport properties. Additionally, we delve into the intricacies of doping graphene to modulate its bandgap—an essential step in fabricating effective field-effect transistors (FETs). By examining various physical and chemical doping techniques thoroughly, we uncover their transformative impacts on electronic parameters and device behavior.

By combining these pivotal aspects of GFET fabrication and analysis, this comprehensive review constructs a holistic reference for the academic and scientific research community. It not only highlights current capabilities and limitations in GFET development workflows but also facilitates the identification of innovation areas to realize the immense promise of graphene-based electronics. Our in-depth investigation serves as an invaluable guide, optimizing the performance of emerging GFET devices while addressing key challenges in their development.

**Figure 2:**
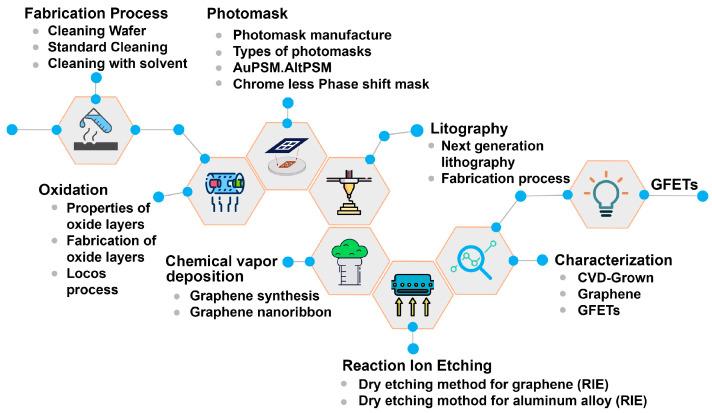
The flowchart encapsulates the sequence of complex steps required to create GFETs discussed in this review, starting from the preparation of the substrate to the final characterization of the completed transistors. Each step is critical to the performance and yield of the GFETs.

In this review, we systematically dissect the GFET fabrication process, as shown in Figure 2. Beginning with synthesis and transfer methods, we critically assess the state-of-the-art techniques that strive to preserve the intrinsic properties of graphene while adapting it to scalable device architectures. The quality of graphene, being the linchpin of device performance, necessitates rigorous evaluation. To this end, we examine a suite of metrology techniques, highlighting their insights into graphene’s crystalline quality, defect density, and uniformity. Moreover, the intrinsic semi-metallic nature of graphene requires a shift in paradigms to induce a controllable bandgap, rendering doping processes pivotal. We scrutinize the effects of various doping methodologies, from chemical to physical adsorption and substrate-induced to electrostatic doping, detailing how each method influences graphene’s electronic, structural, and quantum transport properties. This review aims to provide a comprehensive resource for the graphene research community, facilitating an understanding of the critical aspects of GFET fabrication and providing guidance for future innovations. Navigating these complex facets, we aim to articulate a clear path toward realizing high-performance graphene-based electronic systems.

## 2. Graphene Field-Effect Transistors

GFETs represent a groundbreaking advancement in nanoelectronics, leveraging graphene’s exceptional properties to revolutionize transistor technology potentially. Graphene exhibits extraordinary electrical, thermal, and mechanical characteristics, making it an ideal candidate for transistor applications [7]. The core principle of a GFET lies in using graphene as the channel material through which electric charge flows. Figure 1a illustrates a typical structure of GFETs. Unlike traditional silicon-based transistors, GFETs exploit graphene’s high electron mobility and ambipolar electric field effect. This results in transistors operating at higher frequencies and potentially consuming less power. The high carrier mobility in graphene allows for faster charge transport, contributing to the superior speed of GFETs compared to their silicon counterparts. Additionally, because graphene sheets are so adaptable, they may be used to create transistors on a variety of surfaces, including flexible materials. This creates new opportunities for innovative applications of portable and wearable electronics. The potential for miniaturization beyond the limits of silicon-based technologies is another compelling aspect of GFETs, aligning with the ongoing trend of device miniaturization in electronics.

However, the development of GFETs also faces challenges, particularly in controlling the bandgap in graphene and achieving large-scale, uniform production. Despite these hurdles, ongoing research and advancements continue to enhance the viability of graphene-based transistors, making them a promising candidate for future electronics. Their development significantly impacts the semiconductor industry and opens new avenues in various fields, including high-speed computing, sensors, and advanced communication technologies.

In the FET domain, the key performance indicators for recently described FETs using 2D materials are collated and benchmarked in Table 1 [8,9,10,11,12,13,14]. Graphene shows a very high field-effect mobility (μFE) of 30,000 cm^2^/Vs, as seen in real device simulations, which far exceeds other materials. This extremely fast charge transport is highly advantageous for high-frequency and high-speed applications. The innovative silicon-on-insulator few-layered graphene structure enables a substantial 7 × 10^8^ on/off ratio improvement compared to its inherent theoretical limit of around 10, which is crucial for digital applications where distinct switching between states is necessary. Moreover, with a relatively low subthreshold swing (SS) of 61.03 mV/dec and drain-induced barrier lowering (DIBL) of 25.95 mV/V, GFETs indicate a strong ability to control short-channel effects. These merits showcase graphene’s potential to outperform other materials in FET applications, provided that challenges related to its bandgap are effectively managed.

The recent advancements in the synthesis of large-area, high-quality graphene films have markedly accelerated research in the realm of graphene-based devices [15,16,17]. Its two-dimensional nature and distinctive band structure significantly enhance graphene’s potential in various technological sectors. These characteristics are pivotal in developing advanced applications, particularly in sophisticated electronics. The fast carrier mobility inherent in graphene facilitates high-speed data transmission, making it an invaluable material for cutting-edge electronic devices. Additionally, graphene’s mechanical flexibility opens possibilities for its integration into various form factors, ranging from bendable electronics to innovative composite materials. This adaptability is crucial in the ever-evolving landscape of material science and technology, where versatility and resilience are essential. Moreover, graphene’s unique carbon atom arrangement lends itself to robust and lightweight applications. This aspect is particularly beneficial in creating durable yet light composites, which are essential in sectors like the aerospace and automotive industries. In energy storage systems, graphene’s properties enable the development of more efficient and higher-capacity batteries and supercapacitors, addressing the growing demand for sustainable and reliable energy solutions. The intersection of these properties in a single material positions graphene as a cornerstone for future advancements across a broad spectrum of fields. Its role in driving innovation and enhancing the capabilities of existing technologies cannot be overstated, marking it as a critical material in shaping the future of technology and engineering [18].

Graphene’s hexagonal lattice is not merely a marvel of structural chemistry but the foundation of its impressive physical properties. The stability imparted by this two-dimensional network of bonded carbon atoms is just the beginning. Measuring a mere 0.335 nanometers in thickness, a graphene layer is the ultimate in thin-film technology—yet its scalability is profound. Graphene shows promise for use in applications requiring atomic-scale thinness and macroscale coverage, as it can support a stack of more than 1.5 million layers within a millimeter’s breadth. Consider that within the breadth of a millimeter, one could fit a stack of graphene layers totaling over a million and a half in number, a testament to its potential for use in applications demanding layers of atomic-scale thinness combined with macroscale area coverage [19]. Graphene’s electrical properties are extraordinary and represent a paradigm shift in material science. At the heart of its exceptional electrical characteristics is the massless Dirac fermion-like behavior of charge carriers, which endows graphene with an exceptionally high electron mobility—over 200,000 cm^2^/Vs at room temperature, surpassing silicon by nearly two orders of magnitude. This high mobility is maintained even at a low carrier concentration, a critical trait for high-speed electronic devices. Furthermore, graphene’s intrinsic conductivity is remarkably resilient against temperature variations, maintaining performance over a wide thermal range. However, the material’s zero bandgap means that pristine graphene cannot be switched off like conventional semiconductors, prompting extensive research into bandgap engineering techniques such as substrate-induced bandgap opening or bilayer graphene structures. Additionally, graphene’s ambipolar electric field effect modulates the conductivity over an extensive range, permitting the same material to carry either holes or electrons as charge carriers. This combination of high mobility, stability, and flexibility in charge carrier control positions graphene as a revolutionary material for next-generation electronic components, ranging from transistors to interconnects and flexible electronic applications.

The electrical conductivity of graphene sets new precedents, surpassing traditional benchmarks with its minimal resistive losses and electron mobility that significantly exceeds that of classical semiconductors like silicon by an order of magnitude. Notably, graphene’s conduction efficiency has advanced to a regime where energy dissipation can be quantified in attojoules per switching operation, heralding a new era of ultra-low power electronic devices [20]. Moreover, superconductivity, previously a conjectural attribute for graphene, has been realized under certain conditions, such as when doped or when it is in twisted bilayer configurations. This establishes graphene as a formidable candidate for disruptive technologies in quantum computing and rapid electronic systems. Graphene’s isotropic nature ensures that its exceptional thermal conductivity is not directionally biased, which starkly contrasts with materials like carbon nanotubes with anisotropic heat conduction properties. This quality enables it to act as an ideal heat spreader, evenly distributing thermal energy and managing hotspots in electronic devices with unparalleled efficiency. Its linear dispersion of Dirac fermions near the K points in the Brillouin zone facilitates a uniform absorption of approximately 2.3% of incident light across a wide spectral range, from the visible to the far infrared, irrespective of the wavelength. This intriguing behavior is attributed to the fine-structure constant, resulting in highly transparent yet conductive material characteristics. Furthermore, graphene exhibits a strong interaction with light that can be tuned through electrical gating, enabling the modulation of its optical absorption. This tunability and its compatibility with silicon-based technologies make graphene a promising candidate for a new generation of optoelectronic devices, including photodetectors, modulators, and transparent conductive electrodes. The interplay between its optical conductivity and nonlinear optical responses also opens avenues for exploring novel photonic applications and understanding light/matter interactions in reduced dimensions. High-efficiency solar cells leveraging graphene’s unique optical properties could lead to thinner, lighter, and more flexible solar panels [19].

Astoundingly, a single gram of graphene possesses the capacity to span an entire football field, courtesy of its astonishingly low areal mass density of merely 0.77 milligrams per square meter. This remarkable attribute renders it exceedingly advantageous for producing transistors where material economy and lightweight characteristics are pivotal [21]. The complex fabrication methodology for devising graphene-based devices encompasses a series of intricate steps, each contributing to the nuanced construction of these advanced components. This process is elucidated in detail within Figure 2, which serves as a cornerstone for the in-depth analysis presented in this comprehensive review.

Graphene’s intrinsic electrical and optoelectronic characteristics have been identified as particularly beneficial for constructing optoelectronic devices [22,23,24,25,26,27]. The pursuit of enhanced material response has led to the innovation of an array of graphene photodetector devices. Among these, devices that integrate a Fabry–Perot microcavity, those that couple graphene with a silicon optical waveguide [28,29], and configurations that employ quantum dots [30] have been noted for their innovative approaches to photodetection. Engineering efficient photodetectors based on large-area GFET becomes crucial for photodetection applications that require the swift and reliable detection of optical signals [31,32]. Such devices leverage the large active areas afforded by graphene films to improve the interaction with light, enhancing their responsivity and operational speed. In this context, the continued development of graphene photodetectors is a pivotal area of research, promising to advance the optoelectronics field and pave the way for groundbreaking applications.

Spintronics, short for spin electronics, is a field of research that focuses on exploiting the intrinsic spin of electrons and their charge for information processing and storage. Graphene has emerged as a promising material for spintronics due to its exceptional electronic and spin transport properties. The combination of spintronics and graphene holds excellent potential for developing faster, more energy-efficient devices, such as spin transistors and memory storage elements, by harnessing the long spin relaxation times and high carrier mobility in graphene. This synergy could lead to novel spin-based technologies with enhanced performance and reduced power consumption [33].

With its remarkable electronic properties, graphene is an ideal platform for exploring electrical analogs to optical phenomena and devices. Inspired by Mie scattering, theoretical and experimental studies have demonstrated that graphene can support phenomena such as electron lensing, resonant scattering, and quasi-bound states by manipulating Dirac-electron waves by circular gated regions acting as quantum dots [34,35]. Additionally, the concept of an electronic Lévy glass has been proposed, where the transmission of electrons in graphene nanoribbons (GNRs) is influenced by electrostatic clusters, leading to a transition from super-diffusive to diffusive transport regimes. These graphene research advancements open new avenues for designing functional electronic metamaterials and devices that mimic optical behaviors, offering potential applications in areas such as electron optics, quantum computing, and advanced sensing technologies [36].

Graphene biosensors represent a cutting-edge intersection of nanotechnology and biomedicine, offering revolutionary possibilities in diagnostics and bioanalysis. Graphene’s properties make it an ideal material for biosensing applications. The core principle behind graphene biosensors is their ability to detect biological molecules with high sensitivity and specificity. This is achieved through the interaction of these molecules with the graphene surface, which results in measurable changes in the material’s electrical properties. These changes are often due to the binding of biomolecules, such as proteins, DNA, or small molecules, to the graphene surface, which alters its conductance. One of the most exciting aspects of graphene biosensors is their potential for the real-time, rapid detection of a wide range of biological markers. This makes them highly suitable for applications in medical diagnostics, environmental monitoring, and even in the food industry for detecting contaminants. Their ability to operate at the nanoscale allows for detecting deficient concentrations of biomolecules, which is crucial for early disease diagnosis and precision medicine.

Moreover, graphene’s adaptability and compatibility with various substrates open possibilities for developing wearable biosensors. Such devices could continuously monitor various health markers, providing valuable data for personalized healthcare. Ongoing research is focused on enhancing the sensitivity and selectivity of graphene biosensors and integrating them into user-friendly, cost-effective devices. Integrating graphene biosensors with other technologies, such as microfluidics and electronic data processing systems, is also a key development area, aiming to create fully integrated biosensing platforms.

In a GFET, a channel of graphene is strategically positioned between two electrodes, accompanied by a gate contact that governs the channel’s electrical behavior, as illustrated in Figure 1a [37,38]. The configuration is designed such that graphene is exposed, enabling the channel surface to be functionalized and facilitate the binding of receptor molecules. In GFETs, functionalizing the graphene channel’s surface is paramount, as it allows for the precise attachment of receptor molecules chosen explicitly for their affinity to a target analyte [39]. This functionalization process is critical: it transforms the GFET from a mere electronic component to a highly selective sensor with the capacity to detect and respond to the presence of single molecules. When these target molecules alight upon the tailored receptors, they initiate a subtle yet significant redistribution of charge carriers in the graphene lattice. This interaction triggers a discernible shift in the electric field within the FET channel, which subsequently modulates the conductivity of graphene. It is this delicate modulation that underscores the GFET’s remarkable sensitivity. The binding event is transduced into an electrical signal, a fluctuation in conductance, which is meticulously measured to deduce the presence of the target molecule [40]. The implications of this technology are profound. By leveraging the singular electrical characteristics of graphene and its surface science, GFETs are poised to advance beyond the conventional boundaries of electronics into the biosensing domain, where the detection and quantification of biological and chemical species are critical. This includes applications ranging from environmental monitoring to health diagnostics, where the demand for high specificity and sensitivity is paramount.

## 3. Fabrication Process

### 3.1. Wafer Cleaning

Wafer cleaning, a process of paramount importance in integrating graphene into semiconductor devices, gains heightened significance due to the extraordinary sensitivity of graphene to surface contaminants [41,42]. The exceptional electrical properties of graphene—properties that outstrip those of conventional semiconductor materials—are easily compromised by adsorbed molecules, which can scatter charge carriers and diminish the material’s intrinsic mobility [43,44]. Thus, the presence of even monolayers of contaminants can lead to significant performance degradation in graphene-based components. The wafer cleaning process is a pivotal component in the semiconductor manufacturing workflow, essential for ensuring the integrity of microelectronic devices [45].

Graphene wafer cleaning is a critical process in the fabrication of graphene-based devices, and it varies significantly depending on the type of substrate used. Each substrate material presents challenges and requires tailored cleaning methods to ensure the integrity and quality of the graphene layer. The choice of the cleaning method directly impacts the electronic properties and performance of the final graphene devices, making the selection of an appropriate cleaning technique crucial for each substrate type. A deep understanding of the interaction between graphene and various substrates under different cleaning conditions is essential for optimizing the fabrication process and achieving high-quality graphene-based electronic devices.

The semiconductor manufacturing process heavily relies on meticulous cleaning techniques to ensure the quality and uniformity of its electronic components, particularly in producing high-quality devices like graphene-coated wafers. The Radio Corporation of America (RCA) clean, a fundamental step in this process, targets the removal of organic residues, particulate matter, and ionic contaminants from the wafer’s surface, thereby maintaining the integrity of the devices [46]. Additionally, using hydrofluoric acid (HF) is crucial for adjusting the surface’s hydrophilicity to prevent large cracks and for the chemical removal of the silicon oxide layer [47,48]. This is complemented by baking steps that improve the adhesion between graphene and the substrate [49], a critical factor for consistent and reliable device performance [50]. Moreover, efficient contaminant removal involves ultrasonic baths with acetone and methanol, where the wafer is sequentially heated, immersed, and rinsed. This process includes megasonic cleaning, which uses cavitation and mechanical vibrations to dislodge particles without expensive chemicals, proving to be an effective method for maintaining the stringent standards required for graphene transistor applications [51,52,53]. Concluding with a wettability test, these steps ensure the elimination of pollutants and uphold the high standards essential for sophisticated electronic component manufacturing. Another Si-Compatible Cleaning Process for Graphene has been developed to solve the problem of the removal of polymer resist residues, which affect electrical properties like Fermi level [54,55] shifting and decreased carrier mobility [56]. The researchers proposed a novel cleaning technique using low-density Inductively Coupled Plasma of argon (Ar) [57]. Unlike conventional treatments like thermal annealing [56,58], electrical current annealing [59,60], and chloroform treatment [49] to remove polymer contaminants that damage graphene’s planar structure, this low-density inductively coupled plasma can remove polymer residues without harming the carbon sp^2^-bonding of few-layer graphene. This method also restores the carrier mobility and charge neutrality point of few-layer graphene to its pristine state [61,62]. It addresses the challenge of unwanted doping caused by the fabrication process and suggests that this method is feasible for graphene device processes compatible with silicon technology [63].

Hexagonal boron nitride (h-BN) has gained significant interest as a substrate material for graphene-based electronic devices [64,65]. However, the presence of organic contaminants introduced during standard lithography and substrate transfer processes can adversely affect the performance of these devices. These contaminants can significantly alter the Raman spectrum of h-BN flakes and impact their electronic properties [66]. In addressing this issue, researchers have found that a heat treatment process using an argon/oxygen (Ar/O_2_) atmosphere effectively eliminates organic contaminants [67], including residues of adhesive and photoresist, while preserving the integrity of the h-BN flakes. This purification process results in cleaner h-BN substrates that are more conducive to developing high-quality graphene devices. Preliminary electrical transport measurements on these cleaned substrates have shown encouraging results, indicating high carrier mobilities and the presence of undoped graphene, which are crucial factors for optimal device performance [68].

### 3.2. Graphene Synthesis

Chemical Vapor Deposition (CVD) is a pivotal method in materials science, enabling the production of thin films, coatings, and advanced materials. In this process, volatile precursors are introduced into a reaction chamber, where they flow towards a substrate. Upon reaching the substrate’s surface, these precursors chemisorb, leading to a series of chemical reactions. These reactions might involve decompositions, interactions with the substrate, or reactions with other adsorbed species. The by-products or unreacted precursors then desorb from the substrate, carried away by the carrier gas.

Additionally, diffusion can occur, especially in processes involving alloys or compound films, where atoms or molecules might diffuse into the growing film or the substrate. Ultimately, these surface reactions result in the formation of a thin film or coating on the substrate. The CVD process is highly influenced by factors such as temperature, pressure, the flow rate of gases, and the chemistry of the precursors. Different variants of CVD, such as Thermal CVD, Plasma-enhanced CVD, Metal-organic CVD, and Atomic Layer Deposition (ALD), cater to diverse applications by utilizing distinct energy sources and reaction conditions. CVD technology, thus, offers a controlled and tailored approach for depositing materials onto various substrates, playing a fundamental role in applications ranging from electronics to protective coatings.

**Figure 3:**
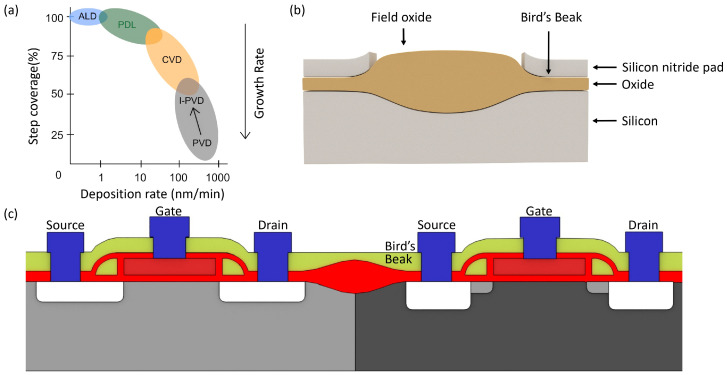
(**a**) Compared different oxide growth techniques for graphene. The vertical axis step coverage shows how well the deposition process can coat the sides and bottom of the features. ALD provides excellent step coverage but at the lowest deposition rates. In CVD, the deposition rate is higher, but the actual growth rate of the crystalline structure is slower due to factors like nucleation rates, the surface mobility of adatoms, and other surface reactions [72]. (**b**) The presence of the pad oxide leads to a sideways spread of oxide underneath the silicon nitride, resulting in a minor expansion of the oxide at the periphery of the nitride mask [73]. (**c**) An example of the LOCOS process for the lateral isolation of transistors. During wet oxidation, nitrogen from the masking agent and hydrogen react to create ammonia (NH_3_), which can migrate towards the silicon surface and induce nitridation. This resulting nitride layer must be eliminated before the gate oxide deposition, as it functions as a barrier, obstructing the growth of the oxide layer [73].

The concept of CVD for synthesizing graphene is not recent, with early experiments dating back to the 1970s. These early attempts aimed to understand the thermodynamics of monolayer and bilayer graphite growth on specific metal substrates like Ni (111) crystals [69]. The CVD process involves the deposition of carbon atoms on metal surfaces using carbon sources such as CH_4_ gas, methanol, or polymethyl methacrylate (PMMA). The quality of CVD graphene depends on factors like reaction temperature (typically 800–1000 °C) and vacuum levels [70,71]. Afterward, CVD graphene can be transferred to various substrates following the chemical etching of the metal substrate. Several metals, including Cu, Co, Pt, Ru, and Ni, have been used as substrates, with Cu and Ni being the most employed. Cu substrates, in particular, tend to yield a higher proportion of single-layer graphene with larger grain sizes when compared to Ni substrates. Various methods of developing graphene are compared in Figure 3a.

Recent attention has been focused on CVD as it allows for producing large-sized graphene sheets [74,75], ranging from tens of microns to a staggering 30 inches. Moreover, CVD offers the advantage of efficiently controlling the thickness of graphene sheets during growth. Additionally, CVD can accelerate the process of substitutional doping by introducing heteroatoms like boron and nitrogen, thus enabling the functionalization and fine-tuning of graphene transistors. Furthermore, CVD-produced graphene can be patterned using microfabrication techniques, making it highly suitable for high-density electrical circuits and miniature electronic devices [76].

Graphene synthesis involves various CVD processes, each with distinct parameters and conditions. Below, we discuss key aspects of these processes and their influence on graphene production: The synthesis of graphene involves a complex set of processes, each playing a crucial role in determining the final quality of the material. Methane, often used as the carbon source, requires a precise methane-to-hydrogen flow ratio, with hydrogen serving to erode amorphous carbon and enhance material quality, although an excess can degrade graphene [77,78]. Using catalysts, such as iron nanoparticles, nickel foam, and gallium vapor, can significantly modify the production process, sometimes necessitating additional steps to remove catalyst remnants [79,80,81,82]. The physical conditions under which graphene is synthesized, like pressure, temperature, carrier gas, and chamber material, are also vital. Low-pressure CVD is commonly employed, with specific temperature ranges enhancing reaction rates but increasing safety risks and energy costs [79,81,83]. Inert gases such as argon and hydrogen are introduced as carrier gases to facilitate movement within the system and improve surface interactions [51,73]. The choice of quartz as a chamber material is due to its high melting point and chemical inertness, ensuring no interference with the synthesis process [84,85]. Various analytical techniques are employed to analyze and characterize graphene samples, each contributing to a comprehensive understanding of the material’s properties [81,86].

A recent study showed that the cold wall CVD approach enables unparalleled control of process parameters such as gas flow rates, temperature, and pressure. As a result, it may be utilized to examine the underlying surface science involved in graphene nucleation and growth. The research was conducted in a vertical cold wall system made at home that used resistive heating by running a direct current through the substrate. It gave a clear understanding of the usual surface-mediated nucleation and growth mechanism in two-dimensional materials generated by catalytic CVD under conditions desired by the semiconductor industry [87,88].

### 3.3. The Graphene Transfer Process

CVD is an essential, sophisticated multi-step procedure for producing high-quality graphene sheets. It begins with meticulously cleaning metal substrates, such as copper or nickel, to eliminate any impurities hindering graphene growth. In the CVD chamber, methane gas is introduced, and under controlled conditions, carbon atoms from the methane decompose and start forming a graphene lattice directly on the metal surface.

Once the graphene layer is grown, a protective layer of PMMA is applied on top. This layer plays a crucial role in maintaining the integrity of the graphene during the transfer process. The metal substrate with the graphene layer undergoes a copper dissolution process. In this step, the metal (usually copper) is etched away, leaving only the graphene layer adhered to the PMMA. The next step involves rinsing the PMMA/graphene layer to remove any residues from the copper dissolution. This step is vital for ensuring the purity and quality of graphene. The cleaned PMMA/graphene layer is then carefully transferred onto a desired substrate, such as silicon dioxide (SiO_2_). This substrate choice depends on the intended application of the graphene. After the transfer, the final and one of the most critical steps is removing the PMMA layer. This delicate step is to ensure the graphene layer remains intact and undamaged. The PMMA removal often involves using solvents or heat treatments, depending on the specific process parameters. Once the PMMA is successfully removed, a high-quality graphene layer on the chosen substrate remains, ready for various applications.

GNRs offer promising avenues for digital electronics due to their ability to introduce a bandgap, a crucial requirement for transistors in electronic devices. The specific arrangement of atoms at the edges of GNRs significantly influences their electronic properties. Zigzag edges confer metallic properties, while armchair edges can be metallic or semiconducting, depending on the width. Unlike graphene, which lacks a bandgap, GNRs exhibit a semiconducting behavior, making them suitable for transistor applications, potentially leading to faster and more energy-efficient electronic devices. However, achieving precise control over the edge structure and width is challenging, impacting the consistent electronic properties of GNRs. Transferring GNRs without causing defects or degradation remains a technical hurdle in their practical implementation [89].

Furthermore, GNRs have diverse applications beyond transistors. Their sensitivity to environmental changes makes them valuable in sensing applications. Additionally, their nanoscale width renders them suitable for various nanodevices and components. Various synthesis methods, including top-down approaches involving lithography and bottom-up approaches using chemical methods, have been employed to produce GNRs. Despite the challenges, harnessing the unique electronic properties of GNRs holds great potential for advancing the field of nanoelectronics and sensing technologies. GNRs are a fascinating graphene derivative with properties that make them attractive for future nanoelectronic devices. Their confinement and edge structure introduce semiconducting behavior, absent in the parent graphene material.

Oxide in GFETs plays a crucial role in the fabrication of GFETs, encompassing the formation of insulating layers, acting as a barrier between the gate electrode and the graphene channel, providing an adhesive surface for graphene, safeguarding graphene from environmental influences, and enabling controlled doping to influence GFET performance.

Controlling the interface between graphene and oxide materials is paramount in the manufacturing process of graphene-based electronic devices, such as GFETs. This involves meticulously adjusting the chemical composition and structural characteristics at the graphene/oxide interface, significantly impacting the device’s electrical conductivity and carrier dynamics. Selecting appropriate oxides like SiO_2_, hafnium oxide (HfO_2_), or aluminum oxide (Al_2_O_3_) and precisely controlling their deposition on the graphene surface are the keys to enhancing device performance. This enhancement is achieved by modifying graphene’s electronic properties through doping or chemical functionalization, which involves introducing specific atoms or molecules to alter carrier concentration or type. Thermal processing and mechanical stress are also vital factors that influence the graphene/oxide interface. Procedures such as thermal annealing can help reduce interface defects, refining graphene’s electrical properties. Additionally, the wettability and adhesion between graphene and the oxide layer are crucial for ensuring interface stability, affecting the device’s longevity and reliability.

In GFETs, oxides function as gate dielectrics, interface layers, or encapsulation materials, directly shaping the device’s electrostatic characteristics. They regulate the gate capacitance and gate coupling efficiency, thereby influencing carrier mobility and the on/off ratio of the transistor. High-quality oxides provide a high-k dielectric environment that enhances carrier mobility by mitigating issues like Coulomb scattering and surface phonon interactions, commonly detrimental to the performance of graphene-based devices. Moreover, the oxide and graphene interface serves as a critical zone where charge traps and states can form. These areas can significantly influence key aspects of the FET, such as the Dirac point and hysteresis in its transfer characteristics. Therefore, engineering this interface through the careful selection and deposition of oxide materials is crucial for minimizing extrinsic doping effects and realizing the intrinsic performance potential of GFETs. Oxides also serve as protective layers against environmental degradation, preserving graphene’s intrinsic electronic properties and ensuring the long-term stability of the device. This aspect is particularly vital considering the sensitivity of graphene to environmental factors like moisture and contaminants.

Furthermore, when graphene is grown on a separate substrate, such as copper, using CVD, the oxide layer on the silicon substrate becomes essential for the subsequent transfer of graphene. It provides an ideal surface for graphene to adhere to, ensuring compatibility and strong adhesion between graphene and the substrate. Additionally, these oxide layers function as protective coatings, passivating the underlying graphene from environmental contaminants and moisture, thereby preserving its electronic properties and enhancing long-term stability. Furthermore, oxidation can be strategically employed to introduce controlled doping into the graphene channel, enabling the fine-tuning of carrier concentration for customized transistor performance. Oxidation stands as a foundational step in GFET fabrication, contributing to gate dielectrics, adhesion surfaces for graphene, protective coatings, and controlled doping—each critical for GFET functionality and performance across a broad spectrum of electronic applications.

In summary, in developing GFETs optimized for advanced electronic applications, the choice of oxide, its deposition method, and the quality of the resulting interface are central to achieving enhanced device performance. They dictate critical device parameters like operational stability, carrier mobility, and overall electronic efficiency, making the control of the graphene oxide interface a fundamental aspect of GFET manufacturing [90].

Graphene oxide interface control is essential in graphene transistor fabrication, emphasizing the management of critical interfaces such as graphene/substrate, graphene/dielectric, and graphene/metal contacts. These interfaces play a significant role in the device’s performance. For the graphene/substrate interface, particularly in top-gated FETs, the gate dielectric deposition on graphene is crucial, impacting the device’s electrical properties and intrinsic transport properties of graphene. Methods like direct deposition or chemical functionalization can introduce defects in the graphene lattice, reducing charge carrier mobility and affecting transistor performance [91]. For the graphene/dielectric interface, dielectric deposition is pivotal in determining graphene’s electrical properties, especially carrier mobility. The quality of this interface influences the overall performance of the FET. Variations in dielectric material and deposition techniques can lead to different interface characteristics, impacting the interaction between the dielectric and graphene. This interaction can alter the distribution and scattering of charge carriers, thereby affecting the mobility and conductivity of graphene in the transistor. For the graphene/metal contact interface, the metal’s interaction with graphene forms a potential step due to the creation of an interface dipole layer. This layer results from the difference in work functions between the metal and graphene, leading to charge transfer and the formation of an electric field at the interface. This electric field affects the Fermi level positioning, altering the electronic properties of the GFET. The quality of this contact interface is crucial for efficient charge injection and extraction, impacting the overall device performance.

SiO_2_ plays a pivotal role in GFET fabrication, adhering to a meticulous sequence of steps. The resulting SiO_2_ layer, inspected post-process, serves as a foundational substrate for the subsequent integration of graphene, underpinning the efficacy of GFETs. The oxidation process for silicon substrates is a vital step in GFET fabrication, ensuring the growth of high-quality SiO_2_ layers fundamental to device functionality. The oxidation method chosen, whether dry or wet oxidation, significantly influences the performance and functionality of GFETs, making it a critical aspect of the fabrication process.

In the fabrication process of GFETs, the formation of the oxide layer is crucial, primarily achieved through methods such as silane pyrolysis, Tetraethyl orthosilicate (TEOS) deposition, and H_2_O_2_ combustion to grow SiO_2_ on silicon substrates. Silane pyrolysis involves the thermal decomposition of silane and oxygen at about 400 °C, while TEOS deposition transforms liquid tetraethyl orthosilicate into a gaseous state at 750 °C to form SiO_2_. On the other hand, the H_2_O_2_ combustion process controls the combustion of hydrogen and oxygen gases at temperatures exceeding 500 °C to produce the oxide layer. These different oxidation techniques significantly impact the performance of GFETs. By precisely controlling the thickness and quality of the oxide layer, which provides essential electrical insulation and interface layers, they affect the overall performance and stability of the device.

An intriguing aspect emerges when contemplating attaining a specified oxide layer thickness—namely, the intricacies of oxidation rate and growth rate dynamics within these layers. The oxidation rate hinges upon the efficiency with which oxygen molecules traverse the SiO_2_ layer, whereas the growth rate links intricately to the temporal aspect of the silicon/oxygen reaction. Notably, as the thickness of the SiO_2_ layer escalates, the growth rate tends to diminish. This phenomenon bears profound implications for optimizing GFET fabrication, profoundly influencing the ultimate electrical characteristics of these devices [92].

Furthermore, it is imperative to address dangling connections, encompassing free electrons and holes, manifesting at the silicon-silicon dioxide (Si-SiO_2_) interface. These unpaired bonds give rise to a marginally positively charged domain proximal to the interface. This charge distribution holds potential ramifications for integrated circuits, including GFETs. In response, mitigation strategies are deployed to ensure the functional integrity of GFETs. Notably, implementing wet oxidation, characterized by minimal charge generation during oxidation, and the elevation of oxidation temperatures are effective measures to ameliorate these interface charges. It is imperative to underline that the applicability of wet and dry oxidation methodologies is contingent upon specific electrical requisites inherent to gate oxides within the domain of GFETs.

The Local Oxidation of Silicon (LOCOS) process is an essential technique for isolating transistors in semiconductor devices, particularly useful in the fabrication of GFETs. It involves growing a thick field oxide layer on the exposed silicon surface while areas covered by a thin nitride mask are left unoxidized, thus providing crucial electrical isolation between transistors and preventing the lateral diffusion of dopants. This method is especially significant in GFETs, where surface contamination is a concern, and the field oxide layer acts as a barrier against impurities, contributing to the high-quality electrical properties of the GFETs. In the Very Large-Scale Integration of GFETs, LOCOS helps achieve high packing density and addresses microfabrication challenges like photolithography and etching, which can create irregular surfaces on the graphene substrate. The process uses pad oxide during the oxidation of bare silicon to control dopant dispersion, leading to a bird’s beak-like oxide buildup at the edge of the nitride mask, as shown in Figure 3b,c, influenced by the oxidation duration and thicknesses of the pad oxide and nitride layers. Additionally, LOCOS can lead to the “white ribbon” or “Kooi effect” in wet oxidation processes, necessitating the removal of the nitride layer before gate oxide deposition. Despite these challenges, LOCOS enhances uniformity and resolution in semiconductor manufacturing, making it a viable method for large-scale integration, including in producing high-performance GFETs.

Several alternative isolation techniques have been developed to address the disadvantages of LOCOS. One popular alternative is shallow trench isolation (STI). STI involves etching shallow trenches into the silicon substrate and filling them with an oxide layer [93]. STI produces a very planar surface and can achieve higher packing densities than LOCOS. However, STI is also more complex and expensive to implement than LOCOS. In the context of advanced semiconductor technology, such as the 45 nm technology node, the dimensions of STI trenches typically range from approximately 100 nm (in the case of silicon-on-insulator, or SOI) to 300 nm (for bulk silicon), respectively. This approach represents a fundamental shift in device isolation methods, optimizing space utilization and enhancing overall semiconductor performance.

Another alternative isolation technique is damascene isolation. Damascene isolation involves depositing a metal layer on the silicon substrate and then patterning it to form trenches. The trenches are then filled with an oxide layer. Damascene isolation is a very versatile technique that can be used to produce a variety of isolation structures. However, it is also more complex and expensive to implement than LOCOS.

### 3.4. Photolithography and Electron-Beam Lithography

**Figure 4:**
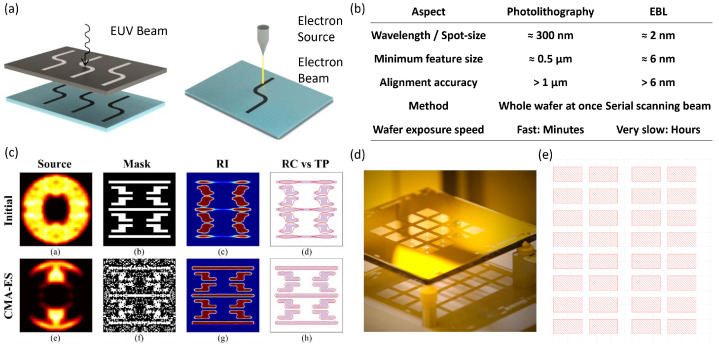
(**a**,**b**) EBL has a higher resolution than photolithography, capable of patterning features well below 10 nanometers. As it is a direct-write technique, this technique is slower and hard to scale, undesired for massive production. The cost for EBL could be higher due to slower throughput and the cost of equipment [94]. (**c**) As feature size decreases to sub-20 nm, photomasks need computational lithography techniques to guarantee image fidelity and process robustness, making designing a mask more complicated and expensive. Adapted with permission from [95] © Optical Society of America. (**d**) An example of a photomask. As the fabrication feature size decreases, photolithography costs increase due to more expensive equipment and masks [96]. (**e**) A GDS file for EBL.

Photolithography and electron-beam lithography (EBL) are pivotal in fabricating and patterning graphene-based devices, each offering distinct advantages and constraints. Figure 4a demonstrates the working principle for EBL and photolithography, and Figure 4b compares the figure of merit of both methods. Photolithography, traditionally used in semiconductor manufacturing, can achieve feature sizes in the sub-10-nanometer range with advanced techniques like extreme ultraviolet (EUV) lithography. This resolution is generally sufficient for a wide range of GFET applications. The primary limitations of photolithography include its diffraction limits and the complexities associated with smaller-scale patterning. On a very small scale, photolithography may struggle to maintain accuracy and uniformity. Additionally, the cost of high-resolution photolithography, especially with EUV lithography, is significant: techniques such as computational lithography shown in Figure 4c and multiple masks with different diffraction corrections shown in Figure 4d need to be implemented, potentially impacting the economic viability of GFET production.

On the other hand, EBL employs a focused beam of electrons to write intricate patterns on an electron-sensitive film directly, excelling in high-resolution fabrication essential for advanced nanoscale graphene devices. It is a maskless operation, and we only need to design a GDS file, as shown in Figure 4e, giving outstanding flexibility. It offers unmatched precision, crucial for the research and development of new graphene technologies, but is generally slower and less suited for mass production, with higher costs due to the complexity and precision of the equipment. The choice between these two techniques hinges on the specific requirements of the graphene device, such as the desired resolution, production scale, and pattern complexity. In some instances, both methods are combined, with photolithography used for overarching large-scale patterning, while EBL is reserved for finer, more complex features. Thus, photolithography and EBL are integral to the graphene fabrication process, each contributing uniquely to the field’s advancement and the realization of innovative graphene-based technologies [97].

Ultraviolet (UV) exposure during photolithography can damage graphene, a material prized for its exceptional electrical and mechanical properties. When UV light shines on graphene, particularly during the patterning steps of device fabrication, the high-energy photons can interact with the carbon atoms in the graphene lattice. This interaction can break carbon/carbon bonds, create defects in vacancies, or disrupt the hexagonal lattice structure. These defects can significantly alter the intrinsic properties of graphene. For instance, they can cause a reduction in electrical conductivity and carrier mobility, both of which are critical for the performance of graphene-based electronic devices. UV-induced defects can increase chemical reactivity at the defect sites, making graphene more susceptible to further chemical degradation or contamination. This degradation is particularly problematic for developing high-performance graphene-based devices, such as FETs, where the integrity of the graphene layer is essential for device functionality. Consequently, the control and minimization of UV exposure during the fabrication process are crucial to maintaining the quality and performance of graphene in various applications.

Electron-beam irradiation on graphene field-effect devices notably impacts their performance in two primary ways. Firstly, there is a significant decrease in the charge neutrality point in substrate-supported graphene, suggesting that the interaction between the energetic electron beam and the substrate leads to graphene doping. Secondly, graphene’s mobility is substantially reduced, and a D peak appears in the Raman spectra, indicating the formation of irradiation-induced defects within the graphene structure. These findings highlight the need for caution when employing techniques such as scanning electron microscopy (SEM), transmission electron microscopy (TEM), and EBL in the imaging and fabrication of graphene devices. Extended exposure to electron beams during these processes can deteriorate the electrical transport properties of graphene. Thus, the study suggests using suspended graphene devices, which are more resistant to radiation, as ideal candidates for applications in radiation-hard electronics [98]. The researchers found that using a low EBL current level results in higher mobility, lower residual carrier density, and a charge neutrality point closer to 0 V, with reduced device-to-device variations. The study revealed a correlation between the mobility of the final devices and the exposure current level used during the active EBL step, showing that mobility is significantly lower at higher exposure currents. Controlling the EBL exposure current and plasma etch time and using thermal evaporation for metal deposition can minimize the effects of trapped radicals, leading to enhanced electrical characteristics in GFETs [99].

In GFET fabrication, phase-shift masks (PSMs) present a blend of advantages and challenges. On the positive side, the PSM significantly improves resolution and feature definition, a critical aspect in defining the intricate channel lengths and gate structures in GFETs. This enhanced resolution facilitates the miniaturization of device features, which is crucial as GFET technology advances toward higher densities and smaller geometries. The PSM offers a cost-effective alternative to scale down feature sizes, providing a middle ground between conventional photolithography and more advanced, expensive lithography techniques like EUV lithography or EBL. However, this approach is not without its drawbacks. Due to the need for precise phase control, the complexity involved in designing PSMs adds layers of difficulty in mask fabrication and alignment.

Furthermore, PSMs can introduce optical proximity effects, necessitating advanced software and modeling to predict and mitigate deviations in the pattern caused by diffraction and interference. Furthermore, while the PSM extends the capabilities of optical lithography, it remains subject to the fundamental diffraction limits of light, which may pose constraints as GFET fabrication pushes into the realm of nanoscale dimensions. Therefore, while the PSM is a significant step forward in GFET manufacturing, balancing its benefits with the inherent complexities and limitations is essential for optimal device performance.

The shift to EUV lithography, with its 13.5 nm wavelength, is a pivotal development for GFET manufacturing. This transition is driven by the need for smaller feature sizes and higher resolution, which is crucial for GFET fabrication. EUV lithography’s higher resolution, essential for the fine features in GFETs, promises improved performance through better gate length and channel width control. However, operating EUV lithography in a vacuum, necessitated by EUV light’s strong absorption by materials like glass, poses challenges. It requires using mirrors and reflective photomasks instead of conventional optical lenses and transparent glass, fundamentally altering the lithography infrastructure [100]. Although EUV lithography offers significant benefits for GFETs, the industry continues to grapple with its adoption due to technical and financial constraints. As a result, enhanced conventional lithography techniques, like phase-shift masks and immersion lithography, remain in use. Operating at a 193 nm wavelength, these methods have been optimized to achieve features as small as 32 nm [15]. While they do not match EUV’s resolution, they currently provide a practical solution for GFET fabrication. EUV lithography is seen as the next step for high-performance GFETs, but due to existing technological constraints, advanced conventional lithography remains essential. As these barriers are tackled by the industry, the complete capabilities of EUV lithography in improving GFETs and other semiconductor devices will be unlocked, representing a notable progression in this field [101].

Patterning graphene sheets, a crucial process in exploiting their unique properties for technological applications, involves various sophisticated techniques. One prominent method is lithography, especially EBL, which allows for high-precision patterning by focusing a beam of electrons to etch desired shapes into the graphene. Another widely used technique is CVD [102], where graphene is grown on metal substrates in specific patterns and later transferred to other surfaces. Photolithography, which uses light to transfer a geometric pattern onto a substrate, is also employed for patterning graphene. A more direct approach is focused ion beams, which can etch away parts of the graphene sheet to create intricate patterns. Lastly, chemical patterning, involving reactive gases like oxygen plasma, selectively removes graphene to achieve the desired pattern. Each method offers a unique balance of precision, scalability, and compatibility with different substrates, opening up many possibilities for graphene-based technologies [103].

The most prevalent method is photolithography, which combines the principles of lithography and etching. It starts with applying a light-sensitive material known as photoresist onto the graphene surface. The photoresist is then selectively exposed to light through a patterned mask, altering solubility. The subsequent development step washes away either the exposed or unexposed photoresist, depending on the type of resist used, thereby revealing a latent pattern on the graphene. The final step involves etching, typically using oxygen plasma, to remove the unprotected graphene, resulting in the desired pattern. This method is widely favored for its precision, efficiency, and scalability balance.

Another advanced technique utilizes block copolymers, molecules that spontaneously form highly regular and predictable patterns. By coating graphene with a thin layer of these copolymers and inducing thermal annealing, the copolymers self-assemble into a nanoscale pattern. Then, this pattern is transferred onto the graphene layer beneath, often using an etching process similar to photolithography. The self-assembly characteristic of block copolymers makes this method particularly valuable for creating uniform, repetitive patterns at the nanoscale.

Nanoimprint lithography offers another approach. In this method, a prepatterned mold is physically pressed onto the graphene, imprinting its pattern onto the surface. Once the mold is removed, the graphene retains the transferred pattern. This technique is celebrated for its high resolution and throughput, making it suitable for large-scale production where pattern uniformity is crucial.

EBL is the method of choice for applications requiring extremely fine patterns. EBL involves focusing a beam of electrons to intricately draw custom patterns directly onto a resist-coated graphene surface. While EBL offers unmatched precision and pattern complexity, it is more time-consuming and costly than other methods, making it ideal for specialized applications where detail and accuracy are paramount.

Chemical patterning represents a different paradigm, focusing on modifying the graphene’s chemical properties. Specific areas of the graphene sheet are treated with reactive chemicals, creating patterns by altering the electrical or optical properties of these areas. This method directly modifies graphene’s properties, opening avenues for creating functionalized surfaces for sensors, electronics, and other advanced applications.

Patterning graphene is crucial in fabricating electronic devices, sensors, and various nanoscale applications. Each method has its advantages and limitations in terms of resolution, cost, and scalability, and the choice of method often depends on the application’s specific requirements.

**Figure 5:**
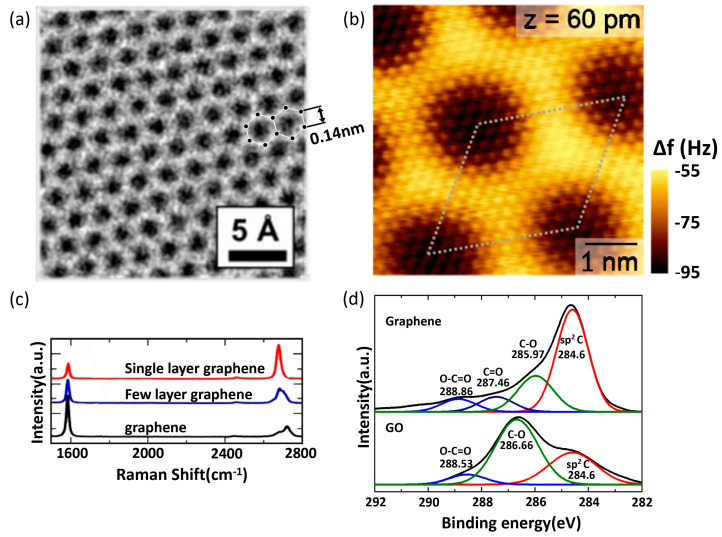
(**a**) TEM image of a graphene sheet illustrating the crystalline lattice (bond length ~0.14 nm). Reprinted with permission from [110]. (**b**) AFM image of a graphene sheet, the bright sections are carbon, and the black sections indicate holes; reprinted with permission from [111]. (**c**) Single-layer graphene exhibits distinctive peaks, including the G peak related to the in-plane vibrations of sp^2^-bonded carbon atoms and the 2D peak, a second-order two-phonon process. The 2D peak is critical as its shape and position can indicate the number of layers in the graphene. In single-layer graphene, this peak is sharp and symmetrical. As more layers are added, the 2D peak becomes broader and shifts. The G peak also shifts higher. Graphite exhibits a broader and more complex 2D peak due to interactions between the multiple layers of graphene in its structure [112]. (**d**) XPS spectra of graphene oxide and graphene. Reprinted with permission from [113].

Graphene Characterization Metrologies are essential for understanding and exploiting this material’s unique properties. One of the primary methods is Raman spectroscopy, as shown in Figure 5c, which provides a rich characterization of graphene, with each peak in its spectrum offering insights into different aspects of its structure and properties. The G peak, appearing around 1580 cm^−1^, is a fundamental feature representing the in-plane vibrations of sp^2^-bonded carbon atoms and is indicative of graphitic content. It is sensitive to the number of graphene layers, strain, and doping. The 2D peak, or G′ peak, located at approximately 2700 cm^−1^, is a second-order overtone of the D peak and is crucial for determining the layer thickness and stacking order in graphene, with its shape and intensity varying noticeably with the number of layers. The D peak, found around 1350 cm^−1^, signals the presence of defects or disorders in the graphene lattice, such as vacancies or edges, and its intensity ratio with the G peak (I_D_/I_G_) is used to evaluate the graphene quality. The D′ peak, near 1620 cm^−1^, is also linked to defects and often accompanies the D peak. Another peak, the G* peak, is a weaker feature around 2450 cm^−1^, reflecting combination modes, varying electronic properties, and doping levels. Lastly, the D + D‘ peak, at approximately 2950 cm^−1^, is another indicator of defects, particularly in heavily defective graphene. Overall, the Raman spectrum of graphene is a valuable tool for assessing its quality, layer number, electronic properties, mechanical strain, and doping, which is critical for its application in various fields [104,105,106,107,108,109]. Atomic force microscopy (AFM), as shown in Figure 5b, is another crucial technique, offering topographical mapping at the nanoscale and enabling the measurement of graphene’s thickness and surface roughness. SEM and TEM, as shown in Figure 5a, are widely used for analyzing graphene’s morphology and crystalline structure. These methods provide high-resolution images, revealing details about layer orientations and edge structures. X-ray photoelectron spectroscopy (XPS), as shown in Figure 5d, is employed to investigate the chemical composition and electronic states, essential for understanding graphene’s chemical properties and verifying the presence of functional groups or dopants. Electrical characterization methods, such as four-point probe measurements, are employed to assess graphene’s conductivity and carrier mobility, which are critical for electronic applications. Together, these metrologies form a comprehensive toolkit for probing the multifaceted properties of graphene, enabling researchers and engineers to tailor its characteristics for a wide range of applications.

### 3.5. GFET Fabrication

The fabrication process began by using a prefabricated silicon wafer. This wafer was specifically chosen for its 50 µm thick silicon oxide layer, which is an ideal substrate for graphene transfer. Initially supported by a polymer, the graphene was carefully transferred onto the SiO_2_/Si substrate. This transfer was accomplished using a water-based process, which is gentle yet effective in maintaining the integrity of the graphene layer. Once transferred, the graphene underwent several post-processing steps, including drying, baking, vacuum treatment, and cleaning. It is important to note that the transfer process is highly delicate and prone to the unintended stacking of the graphene layers, which can significantly affect the material’s electronic properties.

The next phase of the fabrication process involved EBL. This technique was critical for writing global markers essential for alignment purposes. The process began with applying PMMA, a resist material, onto the substrate. This step was followed by baking to solidify the PMMA layer and then developing it to create the desired pattern. The EBL technique was precise enough for subsequent electron beam deposition.

A thicker layer of PMMA was used for the etching process, which is crucial for creating the desired features on the graphene layer with high accuracy. After the etching, the PMMA was carefully removed to leave the etched graphene structure behind. The final step in the fabrication process involved creating the contact pads essential for electrical measurements. The first device iteration used pure gold for these pads, while a subsequent design incorporated a titanium/gold (Ti/Au) combination. This change was complemented by using a different PMMA variant for the etching process, tailoring the fabrication to the specific requirements of the device design.

**Figure 6:**
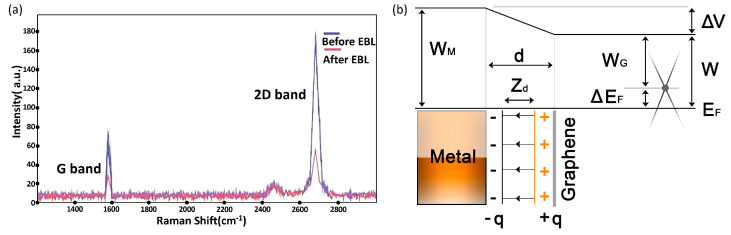
(**a**) Raman spectroscopy of graphene on SiO_2_. The 2D band’s dominance over the G band confirms monolayer graphene. Post electron beam exposure, a decline in the 2D peak indicates the doping effect. (**b**) Schematic representation of the band structure at the graphene metal interface. Here, d denotes the equilibrium separation distance.

Raman spectroscopy played a pivotal role in characterizing the graphene layers. After transferring the graphene onto the SiO_2_ substrate, Raman spectroscopy was employed to investigate its properties. This technique is susceptible to the number of graphene layers and structural imperfections. One can determine the number of layers in the graphene sheet by analyzing the intensities of the G and 2D bands in the Raman spectrum. For instance, in single-layer graphene, the 2D band is more intense than the G band. However, this relationship is reversed in multilayer graphene. As shown in Figure 6a, the Raman spectra of graphene before and after electron beam exposure were compared. The dominant 2D band in the pre-exposure spectrum confirmed the monolayer nature of the graphene. However, after exposure to the electron beam, a noticeable decrease in the 2D peak intensity was observed, indicative of doping effects. Doping alters graphene’s electronic properties by shifting its chemical potential away from the Dirac point, affecting the recombination probabilities of excited charge carriers. This change is critical in tuning the graphene’s electrical properties for various applications.

**Figure 7:**
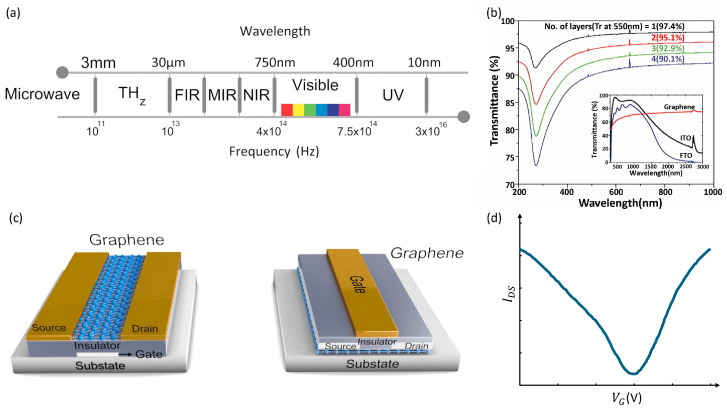
(**a**) Optical spectrum from terahertz to UV. Reprinted with permission from [116]. (**b**) UV/visible spectra of CVD graphene films with one to four layers. Reprinted with permission from [117]. (**c**) Schematic diagrams of two typical GFET structures: bottom gate top contact structure and top gate top contact structure. (**d**) Typical transfer curve (I_DS_ ∼ V_G_) of a GFET that shows ambipolar behavior. Reprinted with permission from [116].

GFETs: A typical FET comprises three electrodes: source, drain, and gate, a channel region connecting the source and drain, and a dielectric layer that isolates the gate from the channel [114]. Various studies have employed two principal FET geometries, bottom-gate and top-gate architectures, as shown in Figure 7c. The gate voltage (V_G_) application between the gate and the source plays a pivotal role in controlling the conductivity of graphene channels [115]. Some studies have even introduced devices with twin gates, combining top and bottom gates within a single device, for in-depth investigations into device physics.

GFETs often exhibit ambipolar behavior in their transfer characteristics (channel current I_DS_ vs. V_G_), as shown in Figure 7d, because graphene is a semimetal with a zero bandgap. The V_G_ can be precisely employed to alter the channel’s carrier type and density. Notably, near the Dirac point, also known as the charge-neutral point where carrier density approximates zero, graphene’s conductivity reaches its lowest value [118]. Depending on the V_G_, a nor p-channel can form on either side of the Dirac point. The position of the Dirac point in a transfer curve is influenced by the difference in work functions between the gate and graphene, the type and density of charges at the interface between graphene and the substrate, and impurity-induced doping levels within the graphene [119,120]. Consequently, GFETs show great promise as transducers for various sensors, including photodetectors, biosensors, and chemical sensors, owing to the devices’ high sensitivity to potential and charge density changes at graphene channels or interfaces.

One of the most compelling features of GFETs is their ultrahigh carrier mobility, exceeding 10^6^ cm^2^/Vs at low temperatures. This mobility is more than ten times greater than in indium phosphide (InP) high-electron-mobility transistors and two orders of magnitude higher than silicon transistors. Importantly, these high mobilities have been achieved at room temperature [121]. However, it is crucial to note that graphene’s mobilities are highly dependent on factors such as its quality, gate insulator, and supporting substrates due to interactions between graphene and insulators or substrates and the presence of charge traps at or near interfaces. Therefore, enhancing interface qualities can lead to increased GFET mobilities. High mobility is particularly vital for graphene-based photodetectors, including extremely IR-sensitive hybrid GFETs and high-frequency GFET-based photodetectors, as it allows these devices to operate at very high frequencies, surpassing Si MOSFETs and competing favorably with InP or gallium arsenide (GaAs) high-electron-mobility transistors in terms of cut-off frequency [116]. Figure 7a,b show the high working coverage spectrum. For instance, self-aligned GFETs with a 67 nm gate have demonstrated the highest observed cut-off frequency, reaching around 427 GHz, approximately three times higher than Si MOSFETs (150 GHz). Notably, GFET-based photodetectors have exhibited ultrafast photo response up to 40 GHz without degradation, and the theoretically predicted RC-limited bandwidth of these devices reaches up to 640 GHz. These advancements hold significant potential for high-speed optical communications.

However, it is essential to note that the on/off ratios of GFETs are often lower than 20, posing a fundamental challenge in their application in logic devices due to graphene’s inherent zero bandgap [122]. Researchers have explored strategies to open a bandgap in graphene to address this limitation, such as the one-dimensional confinement of graphene (e.g., GNRs or quantum dots), the biasing of graphene bilayers, and chemical modification. However, it is crucial to balance bandgap opening and preserve graphene’s high mobility to enhance the on/off ratio of GFETs effectively.

The Leakage Current in GFETs: The unique band structure of graphene, characterized by zero gap width and a linear energy/momentum relation, influences the gate leakage current in GFETs. This leakage current differs from silicon FETs due to graphene’s distinct electronic properties. The Fowler–Nordheim tunneling current in GFETs linearly depends on the oxide electric field and the square root of temperature, meaning that a higher gate electric field leads to a more significant tunneling current in GFETs than silicon FETs. The difference in the gate leakage current between graphene and silicon FETs is attributed to the two-dimensional nature of graphene. A higher oxide electric field results in a larger tunneling current in GFETs. Consequently, a thicker gate oxide might be necessary to ensure functional GFETs to limit gate leakage current. However, controlling the device’s working temperature might mitigate this requirement [123].

While the preceding sections reviewed critical aspects of GFETs, including their fabrication, characteristics, and applications, original experimental work can offer further insights. As a case study, we fabricated and analyzed GFETs using a combination of EBL, electrical measurements, and Raman spectroscopy. The detailed process and results from these experiments are presented in the following sections, providing first-hand perspectives on the practicalities of graphene transistor design, fabrication, and tuning. Specifically, we investigate the impacts of electron beam exposure, contact metals, and channel dimensions on graphene’s electrical properties and chemical potential. Our analysis of custom-fabricated FETs supplements the theoretical discussions with empirical data, helping validate and expand on the core concepts underlying graphene transistors. The synthesis of review content with original experimental findings highlights the intricacies of tailoring GFETs for practical applications and optimizing their performance.

## 4. GFET Results and Analysis

We have developed two innovative designs of GFETs, each targeting different aspects of graphene’s electrical properties. The first design investigates how fabrication techniques and electron beam exposure affect graphene’s chemical potential. We conducted rigorous testing to ensure the reliability of our results, which led us to explore the resistivity characteristics of graphene sheets in greater depth.

Our first design used a monolayer graphene sheet, pre-cultivated on a polymer and sourced from a reputable supplier. The sheet measures 1 cm × 1 cm, an ideal size for manufacturing multiple devices. According to the supplier, the graphene’s electron mobility on a SiO_2_/Si substrate is 3760 cm^2^/Vs, with a sheet resistance of 450 ± 40 Ω/cm^2^. However, our experimental results showed some variations, likely due to differences in the fabrication process and environmental conditions.

This design features two 40 nm gold contacts on opposite sides of the graphene layer, laid on a 50 nm thick silicon oxide layer atop a silicon substrate. The channel dimensions are 8 µm × 10 µm. The gold film, acting as an acceptor, influences the graphene’s properties, with its impact varying by thickness and deposition duration. We constructed a 10 × 10 device matrix on the graphene, incorporating alignment markers for precision.

We observed that our fabrication method altered the graphene’s chemical potential upon testing. Interestingly, our slot-modulator devices exhibited a resistance nearly ten times higher than our transistors, prompting a thorough investigation into graphene’s resistance properties and subsequent adjustments to the channel dimensions.

For our second FET design, we varied the graphene channel dimensions from 100 µm to 10 µm. This experimentation aimed to gain deeper insights into contact resistance, sheet resistance, and overall conductivity. By tweaking the channel size and integrating different materials with graphene, we sought to understand their effects on resistivity and chemical potential. This approach allowed us to explore the nuanced roles of various factors in graphene-based device performance.

Graphene Transistor Analysis and Adjustments: Graphene transistor measurements revealed p-doping by the metal electrode post-processing. Using a 4-probe setup, we assessed the source/drain current changes in the graphene transistor as the gate voltage varied. Two source measure units (SMUs)were connected to the probe station: one applied an electric field between the source and drain, while the other controlled the graphene’s doping level. For these devices, with a 50 nm silicon oxide layer, post-test findings showed a gate voltage limit of around 25 V ± 5 V. Exceeding this risks oxide breakdown and circuit shorting. Subsequent tests capped the gate voltage at 20 V for device safety.

To determine the I-V relationship of the transistor, a constant voltage of 20 µV was applied from the source to the drain using the first SMU. Concurrently, the second SMU was connected from the gate to the drain. The voltage was swept from 20 V to 20 V, and the corresponding current changes from the source to the drain were recorded. All graphene transistor devices were observed to be p-doped. Gold (Au) adsorbs onto the graphene surface with an equilibrium separation of approximately 3.31 Å. As per Equation (1), Au is expected to induce p-type doping in graphene. It should be noted that the doping effect varied across individual transistor devices.

(1) $$∆E_{F}\left(d\right)=\pm \frac{\sqrt{1+2\alpha D_{0}\left(d-d_{0}\right)|W_{M}-W_{G}-{∆}_{c}\left(d\right)|}-1}{\alpha D_{0}(d-d_{0})}$$

where *α* is a constant that characterizes the interaction, and *D*_0_ is the density of states at the Fermi level. *d*_0_ is the reference separation distance. *W_M_* is the work function of the metal. *W_G_* is the work function of graphene. ∆*c* is a potential difference depending on the distance d [124]. Figure 6b shows the band structure and the abovementioned parameters at the graphene metal interface.

The chemical potential of graphene plays a pivotal role in determining its refractive indices, which has significant implications for optoelectronic applications. It is imperative to strategically position the chemical potential to achieve optimal device performance, especially in terms of rapid modulation and minimized static parasitic power dissipation. A target value of 0.4 eV, specifically at its steepest gradient, has been identified as the ideal point for these purposes. Following the intricate process of graphene transfer, the subsequent steps involved meticulous patterning and the careful deposition of electrodes. Post these procedures, the graphene underwent a p-type doping process, which was essential for modulating its electronic properties. One can ascertain the chemical potential of the doped graphene from its I-V characteristics, a standard method that provides insights into the electronic behavior of the material. Upon analysis, device(a) exhibited a chemical potential of −0.276 eV. Device(b), although similar in fabrication, displayed a slightly different chemical potential of −0.286 eV, highlighting the subtle variations that can arise during fabrication. Interestingly, device(c) demonstrated a neutral chemical potential, registering at 0 eV. These variations underscore the importance of consistent fabrication processes and the inherent challenges in achieving uniform doping across different graphene devices.

In Equation (2) [124], the symbol ℏ stands for the reduced Planck constant, a fundamental constant of nature that plays a crucial role in quantum mechanics, and V_F_ is the Fermi velocity. This constant is a derivative of the original Planck constant, which is central to quantizing energy levels in a system. The term V_F_ represents the Fermi velocity. For graphene, a material renowned for its unique electronic properties, the Fermi velocity is approximately 10^6^ m/s. This value is significant as it underscores the rapid movement of charge carriers within the graphene lattice, contributing to its exceptional conductivity. The gate voltage, denoted by V_g_, is an essential parameter in FETs and similar devices. Our analysis assumes that it aligns with V_Dirac_ at a neutral point of 0 V. This assumption is based on graphene’s unique electronic band structure, where the conduction and valence bands meet at the Dirac point. The terms $\varepsilon_{r}$ and $\varepsilon_{0}$ are pivotal in understanding the dielectric behavior of the oxide layer. Specifically, $\varepsilon_{r}$ is the relative dielectric constant, which provides insights into how a material responds to an external electric field relative to a vacuum. On the other hand, *ε*_0_ is the vacuum dielectric constant, a fundamental constant that describes the strength of electric fields in a vacuum. Lastly, *de* is a parameter that signifies the thickness of the oxide layer. This thickness can influence the overall capacitance and, consequently, the device’s behavior.

By integrating all these parameters, Equation (2) provides a comprehensive method to calculate the chemical potential of the system, offering insights into the electronic behavior of the graphene-based device.

(2) $$\left|\mu_{c}\right|=\hbar V_{F}\sqrt{\pi a_{0}\left|V_{g}-V_{\mathrm{Dirac}}\right|}$$

$$a_{0}=\varepsilon_{r}\varepsilon_{0}/de$$

**Figure 8:**
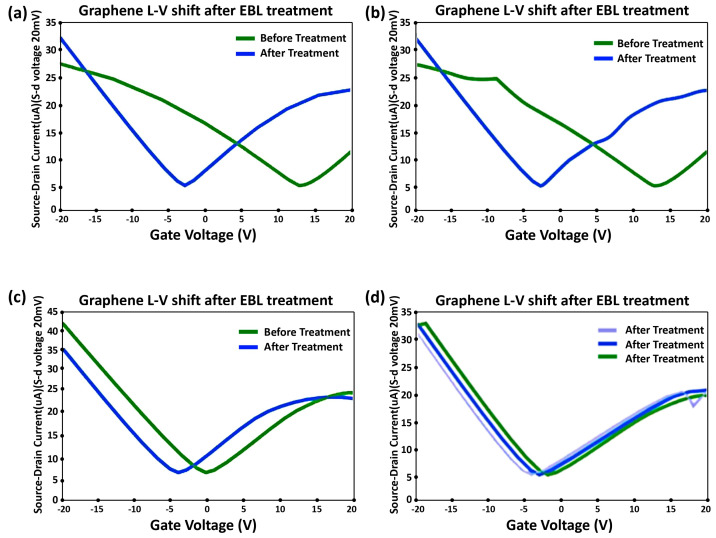
(**a**–**c**) I-V curves of graphene transistor devices before and after EBL. The x-axis denotes the gate voltage, while the y-axis represents the source/drain current. A notable shift from left to right indicates changes in graphene’s electronic properties post-treatment. (**d**) Combined IV curves for all three devices after exposure. The transition from p-type to n-type behavior suggests an increased electron concentration in the graphene. The convergence of all devices to a similar point post-exposure implies electron saturation in the graphene.

The Fermi level of graphene was modulated using electron beam doping. Initial measurements were taken of the graphene transistor’s I-V characteristics and resistance. Subsequently, the graphene channel was exposed to the electron beam to assess the induced changes. The electron beam parameters were set at a voltage of 50 kV, a beam current of 2.0 nA, and a dose of 200 µC/cm^2^. Following the comprehensive exposure of the graphene channel to the electron beam, the transistor’s characteristics were re-measured using the same methodology. Notably, all graphene transistor devices exhibited a shift in their Fermi levels. The previously p-type doped graphene transitioned to n-type doping. Specifically, device(a) in Figure 8a showed a change from approximately 0.409 eV to 0.1326 eV. Device(b) in Figure 8b transitioned from about 0.44 eV to 0.153 eV. Device(c) in Figure 8c demonstrated a shift of 0.133 eV. All devices converged to a similar Fermi level, as illustrated in Figure 8d. When the doping process was repeated on the graphene channel, the chemical potential remained unchanged after the second treatment, suggesting that the electron beam had doped the graphene to its saturation point.

## 5. Future Direction

Quantum capacitance primarily arises from the quantum mechanical properties of materials. It differs from classical capacitance, which is influenced by a material’s physical structure and dielectric characteristics. Quantum capacitance stems from the energy states of electrons within a material and is particularly significant in nanoscale devices where quantum effects become more pronounced. This capacitance type is closely related to the density of states at the Fermi level, representing the number of electronic states available at a specific energy level. This relationship impacts the device’s overall capacitance and performance [125].

**Figure 9:**
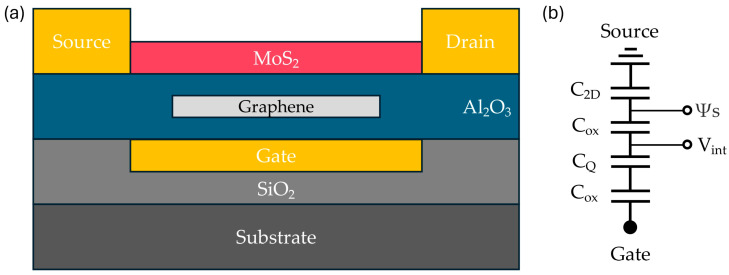
(**a**) Schematic of an NQCFET structure utilizing both MoS_2_ and graphene 2D materials [126]. (**b**) Capacitive components of the gate stack as represented by an equivalent electronic circuit.

Regarding future research for GFETs, exploring quantum capacitance and negative quantum capacitance field-effect transistors (NQCFETs) is pertinent, as shown in Figure 9a. The equivalent electronic circuit illustrating the capacitive components of the gate stack is shown in Figure 9b. NQCFETs operate on the principle of negative quantum capacitance, where adding charge carriers decreases the system’s overall energy. This mechanism may lower the subthreshold swing (the voltage required to increase the current by an order of magnitude in the subthreshold region) below the thermal limit of 60 mV/decade [126]. This limit is a fundamental constraint for conventional FETs. Present research highlights the necessity for a more profound understanding of negative capacitance behaviors, particularly in HfO_2_-based ferroelectrics, commonly used in NC-FETs.

Advancing in this field also involves addressing challenges at very small scales, such as reduced switching speeds, negative bias temperature instability, and hot carrier degradation. These issues are crucial as semiconductor technology continues to miniaturize, with the industry progressing towards below the 5 nm technology node.

## 6. Conclusions

Graphene’s unique crystalline and band structure imparts extraordinary electrical, optical, and mechanical properties. In this comprehensive review, we explored the multifaceted aspects of graphene transistor technology, emphasizing the nuanced balance of parameters necessary for optimizing GFET production. Key findings highlighted the significance of the cleanness of all surfaces, graphene fabrication and transfer, oxide selection and parameters, and characterization in achieving high-performance transistor fabrication. Additionally, we explored the detailed aspects of doping graphene and analyzed how it influences its electronic properties. This investigation revealed how these dopants significantly alter the charge carrier concentration, bandgap, and overall performance of devices.

The potential applications of graphene transistors in high-frequency electronics, flexible devices, and sensors present a promising horizon. However, addressing the inherent limitations, such as the zero-bandgap nature of graphene and the scalability of production methods, remains imperative. Continued research is essential to harness the full potential of graphene transistors, potentially revolutionizing the field of nanoelectronics and material science.

## Figures and Tables

**Table 1:** Comparison of figure of merit of recently studied 2D material FETs. T: the thickness of material; tox: the thickness of dielectric layer; L_CH_: channel length; μFE: field-effect mobility; SS: subthreshold swing; DIBL: drain-induced barrier lowering.

Device Structure	T (nm)	Dielectrics/Tox (nm)	L_CH_ (nm)	μFE (cm^2^/Vs)	On/Off Ratio	SS (mV/dec)	DIBL (mV/V)
Silicon-on-insulator Few-layer Graphene [8]	4	HfO_2_/1	18	30,000	7 × 10^8^	61.03	25.95
Top Gate Few-layer Black Phosphorus [9]	10	Al_2_O_3_/10	20	12	10^2^	90	450
Vertical Short-channel Few-layer MoS_2_ [10]	2.8	HfO_2_/10	8.7	4.92	10^7^	73	100
Double Gate Monolayer MoTe_2_ [11]	0.7	SiO_2_/1.5	7	-	1.44 × 10^7^	77	20
Double Gate Silicene Nanoribbons [12]	2.22	SiO_2_/1.5	10	100	1.15 × 10^5^	66.9	39.8
Back Gate Bilayer WS_2_ [13]	1.95	HfLaO/10	18	31	10^5^	295	300
Double Gate Bilayer WSe_2_ [14]	1.4	HfO_2_/0.037	6	-	2 × 10^6^	76	50

## Data Availability

The data presented in this study are available on request from the corresponding author.
